# Ecology of Porcine Astrovirus Type 3 in a Herd with Associated Neurologic Disease

**DOI:** 10.3390/v12090992

**Published:** 2020-09-07

**Authors:** Gaurav Rawal, Franco Matias Ferreyra, Nubia R. Macedo, Laura K. Bradner, Karen M. Harmon, Grant Allison, Daniel C. L. Linhares, Bailey L. Arruda

**Affiliations:** 1 Veterinary Diagnostic and Production Animal Medicine, College of Veterinary Medicine, Iowa State University, Ames, IA 50011, USA; grawal@iastate.edu (G.R.); francomf@iastate.edu (F.M.F.); nubia@iastate.edu (N.R.M.); lbradner@iastate.edu (L.K.B.); kharmon@iastate.edu (K.M.H.); linhares@iastate.edu (D.C.L.L.); 2 Walcott Veterinary Clinic, Durant St, Walcott, IA 52773, USA; swinedoc2@gmail.com

**Keywords:** astrovirus, porcine astrovirus type 3, neurologic disease, longitudinal study, swine

## Abstract

Astroviruses (AstVs) cause disease in a wide variety of species. Porcine AstVs are highly genetically diverse and conventionally assigned to five genetic lineages (PoAstV1-5). Due to the increasing evidence that porcine astrovirus type 3 (PoAstV3) is a cause of encephalomyelitis in swine and to elucidate important ecologic characteristics, the infection dynamics and environmental distribution of PoAstV3 were investigated in a herd with PoAstV3-associated neurologic disease. Over a 22 week period, the frequency of PoAstV3 fecal shedding varied by pig and age. The peak detection by RT-qPCR of PoAstV3 on fecal swabs (95%; 61 of 64) occurred at 3 weeks of age. The lowest frequency of detection was at 21 weeks of age (4%; 2 of 47); however, the frequency increased to 41% (19 of 46) at the final sampling time point (25 weeks of age). Viremia was rare (0.9%: 4 of 433). Detection in oral fluid was consistent with 75% to 100% of samples positive at each time point. Pens and feeders also had a high rate of detection with a majority of samples positive at a majority of sampling time points. Based on the data presented, PoAstV3 can be consistently detected in the environment with a majority of pigs being infected and a subset intermittently shedding the virus in feces out to 25 weeks of age. These findings suggest the importance of as-yet unidentified risk factors associated with the development of PoAstV3-associated polioencephalomyelitis.

## 1. Introduction

Astroviruses are second only to rotavirus as a cause of gastroenteritis in human infants [1] and were first described in this context in 1975 [2,3,4]. Viruses in the family *Astroviridae* are approximately 28–30 nm in diameter with small projections from the surface accounting for their characteristic star-like appearance by electron microscopy [5]. The family *Astroviridae* is divided into two genera: *Mamastrovirus* (MAstV) and *Avastrovirus* (AAstV), infecting mammalian and avian species, respectively [1].

Porcine AstVs are highly genetically diverse and conventionally assigned to five genetic lineages (PoAstV1-5) which have been further classified into seven genotype species including MAstV3, 22, 24, 26, 27, 31, and 32 [6], potentially reflecting different origins, interspecies transmission, and recombination events [7,8,9,10]. The advent of next-generation sequencing (NGS) methods has provided additional insight into astroviruses and their role in disease. As technology progresses, the spectrum of astrovirus genotype species has increased significantly with concurrent growing evidence that MAstVs are involved in neurologic disease in several mammals including humans, cattle, mink, sheep, and more recently pigs [11,12,13,14,15,16,17,18,19,20]. The pathogenic significance of PoAstVs is not well characterized with the exception of PoAstV3 (MAstV22), which has been recently associated with outbreaks of polioencephalomyelitis in swine in the United States [17,18,19] and Hungary [20].

Due to the increasing evidence that PoAstV3 is a cause of encephalomyelitis in swine [17,18,19] and to better understand the ecology of PoAstV3, four studies were conducted in a growing phase swine production system with an extensive history of neurologic disease due to PoAstV3. These studies included a case-control study, pilot study, clean room study, and a prospective longitudinal investigation.

## 2. Materials and Methods

### 2.1. Study Site

A commercial total confinement growing phase swine production system located in the United States was conveniently selected due to a history of PoAstV3-associated neurologic disease in nursery pigs spanning multiple diagnostic case submissions over months with viral polioencephalomyelitis and concurrent detection of PoAstV3 in central nervous system (CNS) tissue by RT-qPCR in the absence of *Teschovirus A* and *Sapelovirus A*. The herd veterinarian and farm manager reported that 2–10% of nursery pigs developed neurologic clinical signs suggestive of PoAstV3. Suggestive clinical signs were not reported on the sow farm from which these pigs originated or on the finisher site to which these pigs were transported. The production system consisted of a breed-to-wean site from which piglets were transported to a nursery at 3 weeks of age and to a finisher site at 8 weeks of age, where they were housed until marketing at 25 weeks of age. The nursery consisted of 2 barns (Barn-1 and Barn-2), each with 4 rooms. The rooms in Barn-1 contained 10 pens each. The rooms in Barn-2 contained 24 pens each. Each room was stocked with 600–1440 pigs in an all-in/all-out flow. The finishing site consisted of 2 rooms with 20 pens per room. 

### 2.2. Study Designs

**Figure 1:**
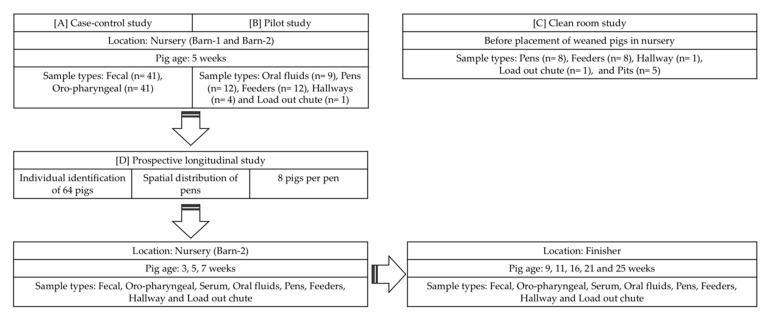
Study design showing sampling time points and sample type collected over the study period. [A] illustrates the barn layout of the case-control study along with sample types used and age of pigs in weeks; [B] illustrates the pilot study to better understand the sensitivity of sample types at the nursery site; [C] illustrates the sample types used for the clean room study; [D] illustrates the sampling points at nursery and finisher sites along with sample types used in the prospective longitudinal study. Arrows are indicative of flow of the study.

The study designs are illustrated in Figure 1. Four studies were completed, which included a (1) case-control study, (2) pilot study, (3) clean room study, and (4) prospective longitudinal study.

### 2.3. Sample Collection and Processing

For all studies, fecal and oropharyngeal swabs were placed in 5 mL polystyrene round-bottom tubes (FALCON, Catalog No. 352054, Corning Incorporated Life Sciences, Tewksbury, MA, USA). Each tube contained 1 mL of 1% phosphate buffered saline (Gibco^TM^, PBS pH 7.4 (1X), Catalog No. 10010023, Grand Island, NY, USA). Sterile polyester-tipped swabs (Puritan^®^, Catalog No. 10805-165, Puritan Medical Products Co., Guilford, ME, USA) were used to collect fecal and oropharyngeal samples. Tubes were prelabelled with pig number and date before sampling. For serum samples, 8.5 mL vacutainer (BD^®^, Catalog No. 367988, Becton Dickinson, Franklin Lakes, NJ, USA) tubes were used. Blood tubes were centrifuged at 3000 rpm for 8 min using a Thermo scientific centrifugation machine, and serum was then poured into a 5 mL polystyrene round-bottom tube (FALCON, Catalog No. 352054, Corning Incorporated Life Sciences, Tewksbury, MA, USA).

Cotton rope was used to collect oral fluid samples [21]. For pen, feeder, hallway, load-out chute, and pit samples, dry pads (Swiffer^®^) were presoaked in 20 mL of 1% PBS per pad and kept in prelabelled bags prior to sampling. For pen samples, five pads (1 per corner and 1 for the center) were used. A single pad was used for the feeder sample. Similarly, for the hallway and load-out chute samples, three pads (1 pad at each end and 1 at the center) were used. A pit sample was collected tying a pad to a clean wooden stick. The oral fluids and pen, feeder, hallway, load-out chute, and pit samples were extracted by squeezing the sample inside a plastic bag. The fluid was then transferred and stored in sterile, polypropylene, 50 mL centrifuge tubes. Samples were stored at −80 °C until testing.

### 2.4. Case-Control Study

The case-control study was done on the nursery farm to determine if clinical signs were associated with the detection of PoAstV3 in fecal and/or oropharyngeal swabs by RT-qPCR. The case-control study was based on clinical evaluation of pigs at 5 weeks of age. The cases were defined as neurologic pigs with one of the following clinical signs: lateral recumbency but cognitively aware, ataxia, astasia, paresis, and/or paralysis. Similarly, controls were selected based on the absence of these neurologic clinical signs as well as no notable respiratory or enteric disease. Nineteen cases were sampled from Barn-1 (*n* = 17; Room-2; Pen-8, Room-3; Pen-5, Room-4; Pen-10) and Barn-2 (*n* = 2; Room-4; Pen-10). Twenty-two controls were sampled from Barn-1 (Room-3; Pen-1, 3, 5, 7, 10). Additionally, four neurologic pigs (two from each barn) were selected for a diagnostic investigation that included gross and histologic evaluation and PoAstV3, *Teschovirus A* and *Sapelovirus A* RT-qPCR, as described previously [18].

### 2.5. Pilot Study

A pilot study was also conducted to understand the detection rate using different sample types including oral fluids, pens, feeders, and hallway samples. These sample types were collected from pens in which there were either clinical pigs (Barn-1: Room-2 and Room-4; Barn-2: Room-4) or spatially distributed pigs (Barn-1: Room-3). One load-out chute sample was taken from Barn-1.

### 2.6. Clean Room Study

The clean room study was performed in Barn-2: Room-4 prior to pig placement. The objective of this study was to illustrate the efficiency of cleaning and disinfection in reducing viral load of PoAstV3. The room was cleaned via pressure washing with cold clean water and disinfected with Synergize^®^ (Neogen Animal Safety, Lexington, KY, USA) as per manufacturer’s recommendation. Pens 1, 5, 9, 12, 13, 17, 21, and 24 were selected based on fixed spatial sampling [22]. Fixed spatial sampling was based on selecting pens equidistant to each other and on alternate sides of the center alleyway over the length of the barn. Samples included pit (*n* = 5), pens (*n* = 8), feeders (*n* = 8), hallway (*n* = 1), and load-out chute (*n* = 1). Pit, Pen, feeder, hallway, and load-out chute samples were collected as described above in sample collection and processing.

### 2.7. Prospective Longitudinal Study

A prospective longitudinal study was done to understand the infection dynamics of PoAstV3 over time using different pig and environmental sample types. A cohort of 64 pigs (8 pigs per pen) were individually identified by ear tag (Allflex TAG SYSTEM) on the day of placement (3 weeks of age) and were spatially distributed in 8 out of 24 pens in the nursery (Barn-2: Room-4). Fecal, oropharyngeal, serum, oral fluids, pens, feeders, hallway, and load-out chute samples were collected on the day of placement (3 weeks of age) and then again at 5 and 7 weeks of age. For the purpose of this study, shedding was measured by detecting PoAstV3 in fecal samples using RT-qPCR. At 8 weeks of age, pigs were moved to a finishing site. The finishing site consisted of 2 rooms with 20 pens per room; tagged pigs were in a single room divided into 8 pens and were sampled at 9, 11, 16, 21, and 25 weeks of age. At 7 weeks of age, two pigs (Pig ID 2 and 25) were selected based on clinical signs (Video S1) for a diagnostic investigation as performed in the case-control study to document the presence of myelitis due to PoAstV3 during the sampling period.

### 2.8. PoAstV3 RT-qPCR

The RT-qPCR methods for PoAstV3 RNA detection were done at the Iowa State University Veterinary Diagnostic Laboratory (ISU-VDL), as previously described [19]. The standard curve is presented in Supplementary Materials file 1.

### 2.9. Statistical Analysis

Statistical analysis was conducted with SAS 9.4 software (SAS Institute Inc., Cary, NC, USA) with a level of significance of 0.05. MS Excel was used to manage RT-qPCR data from four different studies and to extract information regarding detection frequencies, mean, and range of Cq values. The frequency of detection and mean Cq of PoAstV3 by RT-qPCR between sample types (oral fluids, pens, feeders, and pits) in a pilot study and clean room study were analyzed by one-way analysis of variance (ANOVA) followed by Tukey’s multiple comparison test.

*T*-test and Chi-square test were used to compare mean Cq and frequency of detection of fecal and oropharyngeal swabs from the case-control study. The frequency of detection and mean Cq between samples such as fecal, oropharyngeal swabs, and oral fluids were compared using one-way ANOVA followed by Tukey’s multiple comparison test in a prospective longitudinal study. A Chi-square test was used to compare overall detection of PoAstV3 in fecal and oropharyngeal swabs from the prospective longitudinal study. Overall detection rate and mean Cq by RT-qPCR between environmental samples including pens, feeders, hallway, and load-out chute were analyzed using one-way ANOVA followed by Tukey’s multiple comparison test in a prospective longitudinal study.

The odds ratio (OR) was calculated using a two-by-two frequency table, and the 95% confidence interval was calculated using formula upper 95% CI e ^[1n (OR) + 1.96 (1/a + 1/b + 1/c + 1/d)] and lower 95% CI e ^[1n (OR) − 1.96 (1/a + 1/b + 1/c + 1/d)] described by Suzmilas [23], where a = number of cases positive by RT-qPCR +), b = number of cases negative by RT-qPCR (+ −), c = number of controls positive by RT-qPCR (− +), and d = number of controls negative by RT-qPCR (− −). Due to the zero value in the 2 × 2 table (which makes an OR incalculable), 1 was added to each cell in the 2 × 2 table when calculating the odds of detection of PoAstV3 using oropharyngeal samples in pigs with CNS signs compared to clinically healthy pigs.

## 3. Results

### 3.1. Case-Control Study

Detection of PoAstV3 by RT-qPCR in the case-control study is presented in Supplementary Materials file 2. When comparing frequency of detection and Cq values of the RT-qPCR positive fecal swabs, controls had a greater detection rate (3 of 22; mean Cq (range) = 39.10 (38.37–39.45)) compared to that of cases (2 of 19; mean Cq (range) = 33.89 (33.05–34.73)). Likewise, comparing frequency of detection and Cq values of the RT-qPCR positive oropharyngeal swabs, controls had a greater detection rate (3 of 22; mean Cq (range) = 38.45 (37.51–39.59)) than cases (0 of 19). Each positive sample originated from a different individual animal. Using fecal swabs, the odds of detecting PoAstV3 were 0.75 (95% CI: 0.11–5.01) in cases compared to controls. Using oropharyngeal swabs, the odds of detecting PoAstV3 were 0.25 (95% CI: 0.03–2.44) in cases compared to controls.

Significant gross lesions were not observed in any of the pigs evaluated. Histologic lesions consistent with a viral myelitis that included gliosis and perivascular inflammatory infiltrates were present in two of the four cases. PoAstV3 was detected in the CNS by RT-qPCR in the two pigs with histologic lesions and not detected in the two pigs without histologic lesions. Neither *Teschovirus A* nor *Sapelovirus A* were detected in the CNS samples from pigs with or without histologic lesions. PoAstV3 was not detected in samples assayed outside of the CNS including heart, lung, tonsil, spleen, thymus, stomach, kidney, liver, tracheobronchial lymph node, and mesenteric lymph node.

### 3.2. Pilot Study 

PoAstV3 detection frequency and RT-qPCR mean Cq and range by sample type are shown in Table 1. No statistically significant difference in detection frequency or mean Cq of oral fluids, pens, and feeders was found (*p* > 0.05). PoAstV3 was also detected in the hallways and load-out chute at similar Cq values as the other sample types; however, due to the limited number of these samples, statistical analysis was not performed.

### 3.3. Clean Room Study

PoAstV3 was detected in all pit samples (5 of 5; mean Cq (range) = 28.98 (24.85–31.44)), a minority of pen samples (2 of 8; mean Cq (range) = 37.93 (37.84 to 38.03)), and half of the feeder samples (4 of 8; mean Cq (range) = 37.13(35.89 to 38.98)). There was no significant difference in detection frequency of feeder samples compared to pen samples (*p* = 0.27) or feeder samples and pit samples (*p* = 0.062); however, PoAstV3 was detected significantly more in pit samples compared to pen samples (*p* = 0.0080). The mean Cq value of pit samples was significantly lower when compared with the Cq value of pens and feeders (*p* < 0.05). PoAstV3 was not detected in the hallway or load-out chute. 

### 3.4. Prospective Longitudinal Study

PoAstV3 frequency of detection, RT-qPCR mean Cq, and Cq range by sample type over time are presented in Table 2. Fecal swabs had a statistically higher frequency of detection (44%: 190 of 433) in comparison to oropharyngeal swabs (9%: 40 of 433; *p* < 0.0001). Detection of PoAstV3 in oral fluids was consistent over time, ranging from 75% to 100%. No oral fluids were obtained at 3 weeks of age.

Individual animal fecal sample Cq value by age is presented with presence or absence of neurologic signs at 5 weeks of age in Supplementary Materials file 3. At 5 weeks of age, 6 out of 64 pigs (9%) had clinical signs suggestive of PoAstV3. The frequency of PoAstV3 fecal shedding varied over time. The peak of PoAstV3 detection from fecal swabs (95%; 61 of 64) occurred at 3 weeks of age with an average Cq of 31.06, which was the lowest mean Cq value across individual sample types for the duration of the study. Fifteen piglet fecal samples (23%) contained 1.18 × 10^9^ to 2.16 × 10^6^ gc/mL of sample at 3 weeks of age. The next two sampling time points with the highest PoAstV3 frequency of detection was 7 weeks of age (47%; 15 of 53) and 11 weeks of age (88%; 44 of 50). Interestingly, PoAstV3 had the lowest detection rate in fecal samples at 21 weeks of age (4%; 2 of 47); however, the frequency increased to 41% (19 of 46) at the final sampling time point (25 weeks of age).

Individual oropharyngeal sample Cq value by age is presented with the presence or absence of neurologic clinical signs at 5 weeks of age in Supplementary Materials file 4. PoAstV3 was detected in less than or equal to 5% of oropharyngeal samples in 6 out of the 7 sampling time points. The detection of PoAstV3 in the oropharyngeal samples peaked at 3 weeks of age (31%; 20 of 64) and 11 weeks of age (24%; 12 of 50). PoAstV3 viremia was rare (0.92%; 4 of 433). PoAstV3 was detected in the serum of four different pigs at 3, 7, 21, and 25 weeks of age at Cq values that would suggest low viral titers (mean Cq (range): 38.43 (37.61 to 39.33)); (Supplementary Materials file 5).

Gross lesions identified in Pig 2 were extensive and included severe, chronic, fibrinous and fibrosing epicarditis and pericarditis; moderate, fibrinous pleuritis and multifocal pulmonary consolation; moderate, fibrinous polyarthritis; severe bilateral fibrinopurulent rhinitis; fluid-filled large intestine; and sternal lymphadenomegaly. Histologic lesions observed in Pig 2 included a severe lymphohistiocytic and purulent bronchointerstitial pneumonia with fibrinous pleuritis, moderate lymphoplasmacytic cholangiohepatitis, severe fibrinous and fibrosing epicarditis, splenic neutrophilia, mild multifocal lymphohistiocytic interstitial nephritis, and moderate purulent rhinitis. Significant histologic lesions were not observed in the lymph node, tonsil, thymus, small intestine, or large intestine. Histologic lesions were not observed in the cerebrum, cerebellum, brainstem, or spinal cord of Pig 2. PoAstV3, *Teschovirus A* and *Sapelovirus A* were not detected in the spinal cord of Pig 2.

**Figure 2:**
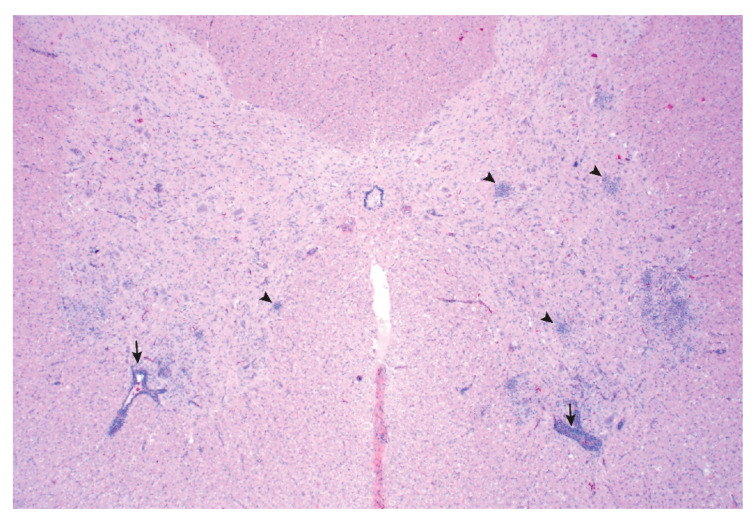
Pig 25 spinal cord: Poliomyelitis characterized by multifocal areas of gliosis (arrowhead) and perivascular inflammatory infiltrates (arrow). The magnification used was 2×.

Grossly, interstitial pneumonia was identified in Pig 25. Multiple histologic lesions were observed outside the CNS of Pig 25 including a severe lymphohistiocytic interstitial pneumonia, moderate lymphocytic myocarditis, and mild lymphoplasmacytic cholangiohepatitis. Significant histologic lesions were not observed in the cerebrum, cerebellum, lymph node, tonsil, thymus, kidney, small intestine, or large intestine. Lesions consistent with a viral myelitis were observed in the cervical, thoracic, and lumbar spinal cord and brainstem of Pig 25 (Figure 2). PoAstV3 was detected by RT-qPCR in the spinal cord of Pig 25 in the absence of *Teschovirus A* and *Sapelovirus A*, as determined by RT-qPCR on CNS tissue. 

The Cq values of individual environmental samples by sampling time point are presented in Supplementary Materials file 6. PoAstV3 was consistently detected by RT-qPCR in different sample types including pens, feeders, hallway, and load-out chute over time (Table 3).

The peak of PoAstV3 detection from pen samples (100%; 8 of 8) occurred at 3 weeks, 7 weeks, and 11 weeks of age. The detection of PoAstV3 in the feeder samples (100%; 8 of 8) peaked at 3 weeks, 7 weeks, and 25 weeks of age. PoAstV3 was commonly detected in hallway, load-out chute, pens, and feeder samples with no significant difference in PoAstV3 detection (*p* = 0.3684) or mean Cq value (*p* = 0.1342) between these sample types (Table 4).

## 4. Discussion

This is the first study that evaluates over time important ecologic characteristics of PoAstV3 using different sample types on a swine farm with a history of associated neurologic disease. As there is limited knowledge concerning the ecology and infection dynamics of *Mamastroviruses* including PoAstV3, this study investigated the detection, distribution, infection dynamics, and shedding of PoAstV3 in a growing phase swine production system with a history of PoAstV3-associated neurologic disease.

The odds of detection of PoAstV3 by RT-qPCR in fecal samples and oropharyngeal swabs was lower in pigs with CNS signs compared to clinically healthy pigs. This could suggest that, at the time of clinical neurologic disease, PoAstV3 is not being shed in feces; however, it may also reflect that there are multiple etiologies that can cause similar clinical signs. Astroviruses, including PoAstV3, are known to infect and be shed by apparently healthy animals [8,19,24,25,26,27]. In the four cases from the case-control study that were selected for a diagnostic investigation, only two cases had lesions consistent with a viral infection in the CNS. PoAstV3 was not detected in any tissue outside the CNS although numerous sample types were assayed. The inconsistent detection of PoAstV3 in tissues, especially enteric tissues, outside the CNS at the time of neurologic disease [18,19,20] could suggest prolonged transport of PoAstV3 from the peripheral nervous system to the CNS. In humans with neurologic astrovirus disease, astrovirus has been reported to be inconsistently detected in feces and infrequently in serum/plasma and cerebrospinal fluid samples [12,28,29,30,31,32].

Others have suggested the respiratory tract as a possible site of infection due to detection of PoAstV3 in 4 of 5 nasal swabs [20]. However, based on the high Cq values and percent positive of oropharyngeal swabs not only in the case-control study but also the longitudinal study, replication of PoAstV3 in the upper respiratory tract appears unlikely. Furthermore, due to the curiosity and rooting behavior of pigs, nasal swabs and the oral cavity commonly contain feces from the environment and may explain the detection of PoAstV3 in the four of five pigs as well as the oropharyngeal swabs in this study given the widespread distribution of PoAstV3 in the environment. During this investigation, PoAstV3 was detected in a large majority of oral fluids as well as pens and feeders.

Following cleaning and disinfection, PoAstV3 was detected less commonly in pens and feeders and at higher Cq values than at other sampling time points. The clean room data would suggest that cleaning and disinfection are efficacious at decreasing the viral load. Continued environmental contamination even after cleaning may be an issue on affected farms. AstVs are resistant to most of the routinely used disinfectants and are extremely stable in the environment [33,34,35]. Further studies are needed to understand the efficacy of disinfectants against PoAstV3.

There was a sharp increase in the detection of PoAstV3 in pen and feeder samples on the day of placement. A direct relationship was seen in the shedding of PoAstV3 in feces with contamination of pens and feeders as detected by RT-qPCR. The contamination of pens and feeders decreased concurrently with the reduction of fecal shedding of PoAstV3 in subsequent sampling time points.

Xiao et al. [10] reported overall detection of PoAstV3 by RT-qPCR in the US as 1.2% (6 of 509) in fecal samples from routine diagnostic cases submitted between 2011 and 2012 to the Iowa State University Veterinary Diagnostic Laboratory. Yet, a recent report found a high frequency of detection of PoAstV3 (65–85%) on three sow farms [19]. These data and the data presented in this report suggest that, while PoAstV3 is infrequently detected in the US swine herd, it can be highly prevalent on farms where it is detected.

Results from the 22 week longitudinal study further support a fecal–oral route of transmission of PoAstV3 which is consistent with what is known for astroviruses [36]. Based on the individual pig data, it appears that pigs shed PoAstV3 intermittently for a long duration of time or can be re-infected. This may be a result of multiple interacting factors including viral attributes, co-infections, previous infections, genetics, stress, and immune status of the individual.

Unlike fecal and environmental samples, PoAstV3 was rarely detected in serum (0.92%; 4 of 433), which is similar to a previous report in which porcine astrovirus was detected in 3.89% (7 of 180) of serum samples assayed [37]. In this study, the viral load in serum was low as determined by Cq values. These data suggest that PoAstV3 viremia is a rare but possible event in the growing phase swine production system with a history of neurologic disease associated with PoAstV3.

## 5. Conclusions

This study investigated multiple ecologic characteristics of PoAstV3 on a single farm with PoAstV3-associated neurologic disease. Based on the data presented in this report, it appears that PoAstV3 is frequently shed in the feces of growing pigs and is distributed throughout the environment on a farm with associated neurologic disease. Given the high frequency of infection and low prevalence of PoAstV3-associated CNS disease in this report, additional investigations could focus on the identification of risk factors associated with the development of neurologic disease in pigs, which will be necessary to prevent and predict disease outbreaks.

## Figures and Tables

**Table 1:** Aggregated PoAstV3 detection frequency and RT-qPCR mean Cq and range in different sample types of the pilot study.

Sample Types	%	Mean Cq ^c^	Mean gc ^d^/mL of Sample ^e^
	(N ^b^)	(Range)	(Range)
Oral fluids	100% ^a^	32.96 ^a^	7.95 × 10^3^
	(9 of 9)	(28.02–38.28)	(2.64 × 10^5^–2.39 × 10^2^)
Pens	75% ^a^	32.23 ^a^	1.60 × 10^4^
	(9 of 12)	(28.66–33.96)	(1.31 × 10^5^–3.94 × 10^3^)
Feeders	100% ^a^	33.86 ^a^	3.94 × 10^3^
	(12 of 12)	(26.60–38.35)	(5.32 × 10^5^–2.39 × 10^2^)
Hallways	100%	32.61	7.95 × 10^3^
	(4 of 4)	(31.87–34.68)	(1.60 × 10^4–^1.96 × 10^3^)
Load-out chute	100%	30.34	6.51 × 10^4^
	(1 of 1)		

^a^ Same letter indicates no statistically significant difference (*p* < 0.05) between the detection rate or mean Cq of sample types. ^b^ N, Number of samples tested by RT-qPCR. ^c^ Cq, Quantification cycle. ^d^ gc, Genomic copies. ^e^ Formula for calculating gc/mL of sample 10^((Cq−45.82)/−3.287)^.

**Table 2:** PoAstV3 detection frequency and RT-qPCR mean Cq and Cq range by sample type over time.

Age (wks ^a^)	Fecal Swabs		Oropharyngeal Swabs		Oral Fluids	
	%	Mean Cq ^c^	%	Mean Cq	%	Mean Cq
	(N ^b^)	(Range)	(N)	(Range)	(N)	(Range)
3	95%	31.06	31%	37.66	NA ^d^	NA ^d^
	(61 of 64)	(16.78–38.88)	(20 of 64)	(32.34–39.27)		
5	17%	35.68	2%	36.22	88%	32.92
	(11 of 64)	(27.07–39.59)	(1 of 64)	-	(7 of 8)	(28.31–36.23)
7	47%	33.63	5%	37.77	100%	31.95
	(28 of 60)	(24.07–39.55)	(3 of 60)	(36.90–38.55)	(8 of 8)	(28.63–34.49)
9	28%	34.85	2%	36.57	100%	31.73
	(15 of 53)	(26.50–38.77)	(1 of 53)	-	(8 of 8)	(27.18–34.25)
11	88%	34.29	24%	37.01	100%	32.20
	(44 of 50)	(27.38–38.54)	(12 of 50)	(34.43–39.94)	(8 of 8)	(30.52–32.50)
16	20%	36.37	2%	36.54	75%	36.14
	(10 of 49)	(30.33–39.57)	(1 of 49)	-	(6 of 8)	(33.36–38.50)
21	4%	37.37	0%	0	75%	35.73
	(2 of 47)	(36.29–38.45)	(0 of 47)	-	(6 of 8)	(33.13–38.28)
25	41%	34.57	4%	37.70	100%	34.23
	(19 of 46)	(30.27–37.85)	(2 of 46)	(37.57–37.84)	(8 of 8)	(33.22–35.48)

^a^ wks, Age in weeks of pigs sampled. ^b^ N, Number of samples tested by RT-qPCR. ^c^ Cq, Quantification cycle. ^d^ NA, Not available.

**Table 3:** PoAstV3 detection frequency and RT-qPCR mean Cq and Cq range by environmental sample type over time.

Age (wks ^a^)	Pens		Feeders		Hallway		Load-Out Chute	
	%	Mean Cq ^c^	%	Mean Cq	%	Mean Cq	%	Mean Cq
	(N ^b^)	(Range)	(N)	(Range)	(N)		(N)	
3	100%	31.01	100%	30.25	100%	31.07	100%	29.10
	(8 of 8)	(29.33–32.43)	(8 of 8)	(27.19–34.70)	(1 of 1)		(1 of 1)	
5	63%	32.83	88%	32.17	100%	29.89	100%	30.28
	(5 of 8)	(30.12–34.77)	(7 of 8)	(27.63–37.83)	(1 of 1)		(1 of 1)	
7	100%	32.13	100%	33.33	100%	29.16	100%	28.49
	(8 of 8)	(27.11–36.57)	(8 of 8)	(29.50–35.34)	(1 of 1)		(1 of 1)	
9	88%	34.14	88%	33.23	100%	33.78	100%	35.37
	(7 of 8)	(29.79–39.40)	(7 of 8)	(27.64–36.46)	(1 of 1)		(1 of 1)	
11	100%	36.31	88%	34.02	100%	32.90	100%	32.90
	(8 of 8)	(33.80–39.97)	(7 of 8)	(33.01–37.02)	(1 of 1)		(1 of 1)	
16	88%	34.69	75%	35.35	100%	35.79	100%	33.41
	(7 of 8)	(32.81–39.42)	(6 of 8)	(33.64–37.59)	(1 of 1)		(1 of 1)	
21	88%	36.49	25%	38.53	100%	32.66	100%	35.75
	(7 of 8)	(34.67–38.20)	(2 of 8)	(38.08–38.97)	(1 of 1)		(1 of 1)	
25	75%	35.63	100%	35.99	100%	35.89	100%	34.00
	(6 of 8)	(34.30–37.77)	(8 of 8)	(33.03–39.74)	(1 of 1)		(1 of 1)	

^a^ wks, Age in weeks of pigs sampled. ^b^ N, Number of samples tested by RT-qPCR. ^c^ Cq, Quantification cycle.

**Table 4:** Aggregated PoAstV3 detection and RT-qPCR mean Cq and Cq range by environmental sample type over time.

Sample Types	%	Mean Cq ^c^	Mean gc ^d^/mL of Sample^e^
	(N ^b^)	(Range)	(Range)
Pens	88% ^a^	33.98 ^a^	3.94 × 10^3^
	(56 of 64)	(27.11–39.97)	(5.32 × 10^5^–5.90 × 10^1^)
Feeders	83% ^a^	33.75 ^a^	3.94 × 10^3^
	(53 of 64)	(27.19–39.74)	(5.32 × 10^5–^5.90 × 10^1^)
Hallway	100% ^a^	32.63 ^a^	7.95 × 10^3^
	(8 of 8)	(29.16–35.89)	(1.31 × 10^5^–9.72 × 10^2^)
Load-out chute	100% ^a^	32.18 ^a^	1.60 × 10^4^
	(8 of 8)	(28.49–35.75)	(1.31 × 10^5^–9.72 × 10^2^)

^a^ No statistically significant difference (*p* < 0.05) between the detection rate or mean Cq of sample types. ^b^ N, Number of samples tested by RT-qPCR. ^c^ Cq, Quantification cycle. ^d^ gc, Genomic copies. ^e^ Formula for calculating gc/mL of sample 10^((Cq−45.82)/−3.287)^.

## Supplementary Materials

The following are available online at https://www.mdpi.com/1999-4915/12/9/992/s1, Supplementary file 1: Standard curve for validation of PoAstV3 RT-qPCR assay, Supplementary file 2: Case-control study detection of PoAstV3 by RT-qPCR, Supplementary file 3: Detection of PoAstV3 by RT-qPCR in fecal swabs by pig over time, Supplementary file 4: Detection of PoAstV3 by RT-qPCR in oropharyngeal swabs by pig over time, Supplementary file 5: Detection of PoAstV3 by RT-qPCR in serum by pig over time, Supplementary file 6: Detection of PoAstV3 by RT-qPCR in pens, feeders, and oral fluids over time, Video S1: Showing clinical signs associated with PoAstV3 in nursery pigs.
