# Supplementary material for: Ecology of Porcine Astrovirus Type 3 in a Herd with Associated Neurologic Disease
Source: Viruses. 2020 Sep 7;12(9):992. doi: 10.3390/v12090992 (PMC7552043; doi:10.3390/v12090992)
Supplement: Supplementary file 1 [file viruses-12-00992-s001.zip › viruses-877412-supplementary-layout/viruses-877412-supplementary.pdf]

Supplementary file 1 Standard curve for validation of PoAstV3 RT-qPCR assay.

CN/mL	Log(CN/mL)	Average
1000	3	36.1
10000	4	32.1
100000	5	29.323775
1000000	6	26.355975
10000000	7	22.9923375
1E+08	8	19.7702875
1E+09	9	16.37405
1E+10	10	13.1501875
1E+11	11	9.1283625

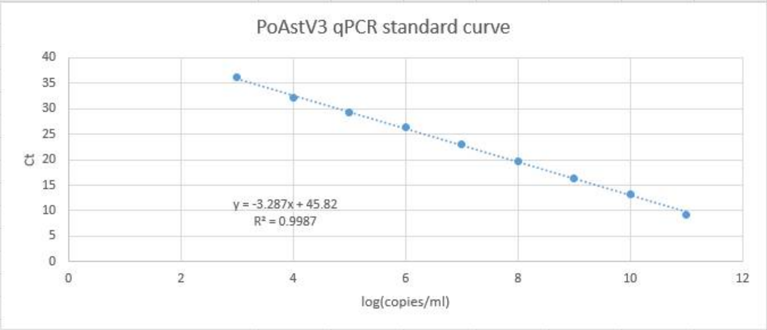

Supplementary file 2 Case-control study detection of PoAstV3 by RT-qPCR.

Pig ID	Group	Feces	Oropharynx
1	Case	ND	ND
2	Case	ND	ND
3	Case	ND	ND
4	Case	ND	ND
5	Case	ND	ND
6	Case	ND	ND
7	Case	33.05	ND
8	Case	ND	ND
9	Case	ND	ND
10	Case	ND	ND
11	Case	ND	ND
12	Case	ND	ND
13	Case	ND	ND
14	Control	ND	ND
15	Control	ND	ND
16	Control	ND	38.26
17	Control	39.16	ND
18	Control	ND	ND
19	Control	ND	ND
20	Control	ND	39.591
21	Control	ND	ND
22	Control	ND	ND
23	Control	ND	37.51
24	Case	ND	ND
25	Case	ND	ND
26	Case	ND	ND
27	Control	ND	ND
28	Control	ND	ND
29	Control	ND	ND
30	Control	ND	ND
31	Control	ND	ND
32	Control	ND	ND
33	Control	ND	ND
34	Control	38.37	ND
35	Control	39.45	ND
36	Control	ND	ND
37	Control	ND	ND
38	Control	ND	ND
39	Case	ND	ND
40	Case	34.7342	ND
41	Case	ND	ND

ND, Not detected following 40 cycles

Supplementary file 3 Detection of PoAstV3 by RT-qPCR in fecal swabs by pig over time.

Pig ID	Clinical signs[a]	Age (in weeks)							
		3	5	7	9	11	16	21	25
1	-	38.49	ND	ND	ND	34.96	38.88	ND	ND
2	+	35.24	ND	ND	NA	NA	NA	NA	NA
3	-	24.56	39.23	38.13	33.23	ND	ND	36.30	ND
4	-	29.12	ND	ND	37.80	28.82	ND	NA	NA
5	-	24.23	ND	ND	28.51	ND	ND	ND	32.84
6	+	23.28	ND	ND	ND	38.13	ND	NA	NA
7	-	20.13	ND	32.49	ND	32.71	ND	ND	32.21
8	-	22.61	ND	ND	35.32	31.21	ND	ND	ND
9	-	22.40	ND	ND	NA	NA	NA	NA	NA
10	+	27.50	ND	NA[c]	NA	NA	NA	NA	NA
11	-	28.61	ND	24.07	ND	35.15	39.18	ND	31.12
12	-	21.23	ND	NA	NA	NA	NA	NA	NA
13	-	34.76	ND	ND	ND	35.32	ND	ND	36.51
14	-	37.10	ND	ND	38.37	35.33	ND	ND	ND
15	-	ND[b]	ND	37.75	NA	NA	NA	NA	NA
16	-	35.07	ND	ND	37.47	34.49	ND	ND	33.04
17	-	38.88	ND	24.49	ND	ND	NA	NA	NA
18	-	33.27	ND	ND	ND	36.28	ND	ND	ND
19	+	29.79	ND	NA	NA	NA	NA	NA	NA
20	-	38.22	ND	30.01	ND	29.50	ND	ND	35.89
21	-	30.49	ND	ND	38.04	35.89	ND	ND	ND
22	-	34.35	ND	ND	NA	NA	NA	NA	NA
23	-	24.64	ND	36.15	37.77	NA	NA	NA	NA
24	-	ND	ND	33.83	26.50	27.39	33.49	ND	31.27
25	+	32.48	32.92	ND	NA	NA	NA	NA	NA
26	-	32.91	ND	ND	ND	33.06	38.64	ND	34.02
27	-	22.12	ND	30.18	ND	37.21	30.34	ND	ND
28	-	29.27	27.07	36.57	38.77	ND	ND	ND	30.49
29	-	34.34	36.96	ND	ND	31.77	39.57	ND	ND
30	-	38.05	ND	34.03	34.49	33.02	ND	ND	ND
31	-	23.88	ND	ND	ND	37.95	ND	ND	ND
32	-	23.16	ND	ND	ND	34.95	ND	38.46	ND
33	-	35.45	ND	ND	ND	36.00	ND	ND	NA
34	-	38.45	ND	ND	38.05	36.37	ND	ND	36.24
35	-	26.95	ND	32.03	ND	36.05	ND	ND	ND
36	-	35.08	ND	ND	ND	35.17	ND	ND	30.28
37	-	16.78	ND	38.89	ND	32.24	ND	ND	ND
38	-	33.05	ND	31.72	ND	35.41	34.20	ND	ND
39	-	30.78	ND	32.67	ND	35.54	ND	ND	ND
40	-	26.86	ND	39.55	ND	36.46	ND	ND	ND
41	-	35.71	ND	28.80	ND	31.46	ND	ND	ND
42	-	32.69	ND	ND	ND	33.51	ND	ND	36.64
43	-	35.88	ND	30.51	ND	35.03	ND	ND	ND
44	-	31.14	ND	ND	ND	34.45	ND	ND	37.12
45	-	38.07	ND	38.65	ND	33.54	ND	ND	ND
46	-	16.88	ND	36.18	ND	ND	ND	ND	37.77
47	-	37.77	ND	36.68	ND	NA	NA	NA	NA
48	-	37.94	ND	36.94	32.19	28.39	ND	ND	ND
49	-	34.25	ND	29.16	ND	36.95	38.69	ND	33.88
50	-	35.44	ND	ND	ND	35.13	ND	ND	37.85
51	-	36.93	ND	26.77	NA	NA	NA	NA	NA
52	-	25.77	ND	37.54	ND	35.53	37.62	ND	ND
53	-	37.54	ND	ND	ND	36.31	ND	ND	ND
54	-	34.09	ND	33.21	ND	34.78	34.20	ND	ND
55	-	36.92	ND	ND	ND	36.30	ND	ND	36.84
56	-	27.30	ND	37.54	NA	NA	NA	NA	NA
57	-	35.32	36.64	ND	ND	33.65	ND	ND	ND
58	-	ND	36.99	ND	ND	38.55	ND	ND	ND
59	-	34.64	39.30	ND	34.98	NA	NA	NA	NA
60	+	23.98	ND	NA	NA	NA	NA	NA	NA
61	-	32.23	35.31	ND	31.24	ND	ND	ND	35.23
62	-	34.88	39.96	ND	ND	35.87	ND	ND	ND
63	-	32.01	29.63	37.12	ND	35.09	ND	ND	37.63
64	-	33.57	38.49	ND	ND	28.13	ND	ND	ND

a Presence (+) or absence (-) of neurologic signs observed at 5 weeks of age.
b ND, Not detected following 40 cycles.
c NA, Not available. Animals had been euthanized or had died.

Supplementary file 4 Detection of PoAstV3 by RT-qPCR in oropharyngeal swabs by pig over time.

Pig ID		Age (in weeks)							
	Clinical signs[a]	3	5	7	9	11	16	21	25
1	-	32.35	ND	ND	ND	ND	ND	ND	ND
2	+	38.59	ND	ND	NA	NA	NA	NA	NA
3	-	38.57	ND	ND	ND	ND	ND	ND	ND
4	-	ND[b]	ND	ND	ND	ND	ND	NA	NA
5	-	38.10	ND	ND	ND	ND	ND	ND	ND
6	+	ND	ND	ND	ND	ND	ND	NA	NA
7	-	ND	ND	ND	ND	ND	ND	ND	ND
8	-	38.01	ND	ND	ND	ND	ND	ND	ND
9	-	35.22	ND	ND	NA	NA	NA	NA	NA
10	+	ND	ND	NA[c]	NA	NA	NA	NA	NA
11	-	ND	ND	36.90	ND	36.93	ND	ND	ND
12	-	ND	ND	NA	NA	NA	NA	NA	NA
13	-	38.91	ND	ND	ND	ND	ND	ND	ND
14	-	ND	ND	37.86	ND	ND	ND	ND	ND
15	-	ND	ND	ND	NA	NA	NA	NA	NA
16	-	ND	ND	ND	ND	ND	ND	ND	ND
17	-	ND	ND	ND	ND	ND	NA	NA	NA
18	-	38.03	ND	ND	ND	36.60	ND	ND	ND
19	+	37.05	ND	NA	NA	NA	NA	NA	NA
20	-	37.95	ND	ND	ND	ND	ND	ND	ND
21	-	38.18	ND	ND	ND	ND	ND	ND	37.84
22	-	38.17	ND	ND	NA	NA	NA	NA	NA
23	-	ND	ND	ND	ND	NA	NA	NA	NA
24	-	ND	ND	ND	ND	ND	ND	ND	ND
25	+	ND	ND	ND	NA	NA	NA	NA	NA
26	-	ND	ND	ND	ND	ND	ND	ND	37.57
27	-	ND	ND	ND	ND	ND	ND	ND	ND
28	-	ND	ND	38.55	ND	37.53	ND	ND	ND
29	-	39.27	ND	ND	ND	ND	ND	ND	ND
30	-	38.13	ND	ND	ND	ND	ND	ND	ND
31	-	ND	ND	ND	ND	ND	ND	ND	ND
32	-	ND	ND	ND	ND	ND	ND	ND	ND
33	-	ND	ND	ND	ND	ND	ND	ND	NA
34	-	ND	ND	ND	ND	ND	ND	ND	ND
35	-	38.55	ND	ND	ND	ND	ND	ND	ND
36	-	ND	ND	ND	ND	ND	ND	ND	ND
37	-	ND	ND	ND	ND	38.30	ND	ND	ND
38	-	38.24	ND	ND	ND	37.83	ND	ND	ND
39	-	ND	ND	ND	ND	34.58	ND	ND	ND
40	-	ND	ND	ND	ND	35.48	ND	ND	ND
41	-	ND	ND	ND	ND	34.44	ND	ND	ND
42	-	35.61	ND	ND	ND	ND	ND	ND	ND
43	-	38.13	ND	ND	ND	39.95	ND	ND	ND
44	-	ND	ND	ND	ND	ND	ND	ND	ND
45	-	ND	ND	ND	ND	ND	ND	ND	ND
46	-	38.12	ND	ND	36.57	37.49	ND	ND	ND
47	-	ND	ND	ND	ND	NA	NA	NA	NA
48	-	38.05	ND	ND	ND	38.32	36.55	ND	ND
49	-	ND	ND	ND	ND	ND	ND	ND	ND
50	-	ND	ND	ND	ND	36.80	ND	ND	ND
51	-	ND	ND	ND	NA	NA	NA	NA	NA
52	-	ND	ND	ND	ND	ND	ND	ND	ND
53	-	ND	ND	ND	ND	ND	ND	ND	ND
54	-	ND	ND	ND	ND	ND	ND	ND	ND
55	-	ND	ND	ND	ND	ND	ND	ND	ND
56	-	ND	ND	ND	NA	NA	NA	NA	NA
57	-	ND	ND	ND	ND	ND	ND	ND	ND
58	-	ND	ND	ND	ND	ND	ND	ND	ND
59	-	ND	ND	ND	ND	ND	ND	ND	ND
60	+	ND	ND	NA	NA	NA	NA	NA	NA
61	-	ND	ND	ND	ND	36.93	ND	ND	ND
62	-	ND	ND	ND	ND	ND	ND	ND	ND
63	-	ND	36.22	ND	ND	ND	ND	ND	ND
64	-	ND	ND	ND	ND	ND	ND	ND	ND

a Presence (+) or absence (-) of neurologic signs observed at 5 weeks of age.
b ND, Not detected following 40 cycles.
c NA, Not available. Animal had been euthanized or had died.

Supplementary file 5 Detection of PoAstV3 by RT-qPCR in serum by pig over time.

Pig ID	Clinical signs[a]	Age (in weeks)							
		3	5	7	9	11	16	21	25
1	-	ND[b]	ND	ND	ND	ND	ND	ND	ND
2	+	ND	ND	ND	NA	NA	NA	NA	NA
3	-	ND	ND	ND	ND	ND	ND	ND	ND
4	-	ND	ND	ND	ND	ND	ND	NA	NA
5	-	ND	ND	ND	ND	ND	ND	ND	ND
6	+	ND	ND	ND	ND	ND	ND	NA	NA
7	-	ND	ND	ND	ND	ND	ND	ND	ND
8	-	ND	ND	ND	ND	ND	ND	ND	ND
9	-	ND	ND	ND	NA	NA	NA	NA	NA
10	+	ND	ND	NA[c]	NA	NA	NA	NA	NA
11	-	ND	ND	39.34	ND	ND	ND	ND	ND
12	-	ND	ND	NA	NA	NA	NA	NA	NA
13	-	ND	ND	ND	ND	ND	ND	ND	ND
14	-	ND	ND	ND	ND	ND	ND	ND	ND
15	-	ND	ND	ND	NA	NA	NA	NA	NA
16	-	ND	ND	ND	ND	ND	ND	ND	ND
17	-	ND	ND	ND	ND	ND	NA	NA	NA
18	-	ND	ND	ND	ND	ND	ND	ND	ND
19	+	ND	ND	NA	NA	NA	NA	NA	NA
20	-	ND	ND	ND	ND	ND	ND	ND	ND
21	-	ND	ND	ND	ND	ND	ND	ND	ND
22	-	ND	ND	ND	NA	NA	NA	NA	NA
23	-	ND	ND	ND	ND	NA	NA	NA	NA
24	-	ND	ND	ND	ND	ND	ND	ND	ND
25	+	ND	ND	ND	NA	NA	NA	NA	NA
26	-	ND	ND	ND	ND	ND	ND	ND	ND
27	-	ND	ND	ND	ND	ND	ND	ND	ND
28	-	ND	ND	ND	ND	ND	ND	ND	ND
29	-	ND	ND	ND	ND	ND	ND	ND	ND
30	-	ND	ND	ND	ND	ND	ND	ND	ND
31	-	ND	ND	ND	ND	ND	ND	ND	ND
32	-	ND	ND	ND	ND	ND	ND	ND	ND
33	-	ND	ND	ND	ND	ND	ND	ND	NA
34	-	ND	ND	ND	ND	ND	ND	ND	ND
35	-	ND	ND	ND	ND	ND	ND	ND	ND
36	-	ND	ND	ND	ND	ND	ND	ND	ND
37	-	ND	ND	ND	ND	ND	ND	ND	ND
38	-	ND	ND	ND	ND	ND	ND	ND	ND
39	-	ND	ND	ND	ND	ND	ND	ND	ND
40	-	ND	ND	ND	ND	ND	ND	ND	ND
41	-	ND	ND	ND	ND	ND	ND	ND	ND
42	-	ND	ND	ND	ND	ND	ND	ND	ND
43	-	ND	ND	ND	ND	ND	ND	ND	37.61
44	-	ND	ND	ND	ND	ND	ND	ND	ND
45	-	ND	ND	ND	ND	ND	ND	ND	ND
46	-	ND	ND	ND	ND	ND	ND	38.41	ND
47	-	ND	ND	ND	ND	NA	NA	NA	NA
48	-	38.36	ND	ND	ND	ND	ND	ND	ND
49	-	ND	ND	ND	ND	ND	ND	ND	ND
50	-	ND	ND	ND	ND	ND	ND	ND	ND
51	-	ND	ND	ND	NA	NA	NA	NA	NA
52	-	ND	ND	ND	ND	ND	ND	ND	ND
53	-	ND	ND	ND	ND	ND	ND	ND	ND
54	-	ND	ND	ND	ND	ND	ND	ND	ND
55	-	ND	ND	ND	ND	ND	ND	ND	ND
56	-	ND	ND	ND	NA	NA	NA	NA	NA
57	-	ND	ND	ND	ND	ND	ND	ND	ND
58	-	ND	ND	ND	ND	ND	ND	ND	ND
59	-	ND	ND	ND	ND	NA	NA	NA	NA
60	+	ND	ND	NA	NA	NA	NA	NA	NA
61	-	ND	ND	ND	ND	ND	ND	ND	ND
62	-	ND	ND	ND	ND	ND	ND	ND	ND
63	-	ND	ND	ND	ND	ND	ND	ND	ND
64	-	ND	ND	ND	ND	ND	ND	ND	ND

a Presence (+) or absence (-) of neurologic signs observed at 5 weeks of age.
b ND, Not detected following 40 cycles.
c NA, Not available. Animals had been euthanized or had died.

Supplementary file 6 Detection of PoAstV3 by RT-qPCR in pens, feeders, and oral fluids over time.

Sample ID	Age (in weeks)							
	3	5	7	9	11	16	21	25
Pen A	30.72	34.77	29.88	ND	33.83	ND	34.67	35.53
Pen B	32.44	ND[b]	34.78	29.79	36.88	33.17	35.49	37.78
Pen C	31.16	ND	27.16	38.46	39.97	32.81	36.76	ND
Pen D	31.12	33.97	31.55	39.40	35.44	33.34	ND	ND
Pen E	32.05	33.67	31.25	34.44	37.18	34.74	35.54	35.09
Pen F	29.34	30.12	32.09	33.01	38.86	33.88	38.20	34.31
Pen G	31.34	ND	33.84	31.87	34.58	39.42	37.85	35.77
Pen H	29.88	31.62	36.57	32.05	33.81	35.53	36.95	35.34
Feeder A	34.71	31.24	29.69	33.74	37.03	37.60	ND	35.81
Feeder B	32.28	34.96	34.12	31.55	ND	34.87	38.08	39.75
Feeder C	31.01	29.18	29.51	34.18	34.25	34.28	38.98	39.51
Feeder D	27.50	ND	31.94	36.42	33.02	35.33	ND	39.60
Feeder E	31.20	37.84	29.9	36.46	33.05	33.64	ND	33.03
Feeder F	30.39	33.26	35.34	ND	33.08	36.38	ND	33.39
Feeder G	27.78	31.08	34.127	32.63	34.28	ND	ND	33.11
Feeder H	27.19	27.64	34.04	27.65	33.45	ND	ND	33.77
Oral fluid A	NA[a]	30.25	33.54	33.27	32.37	38.50	33.13	35.02
Oral fluid B	NA	ND	32.55	29.87	32.57	33.30	33.83	33.23
Oral fluid C	NA	36.23	28.63	32.58	32.37	35.58	36.91	34.62
Oral fluid D	NA	34.19	31.82	34.07	32.58	36.20	37.14	33.64
Oral fluid E	NA	32.71	29.46	34.25	30.52	ND	38.28	35.10
Oral fluid F	NA	33.93	32.37	33.64	32.80	35.11	35.09	33.49
Oral fluid G	NA	34.81	32.73	28.98	32.19	ND	ND	35.48
Oral fluid H	NA	28.31	34.49	27.18	32.32	38.08	ND	33.22
Hallway	31.07	29.89	29.16	33.78	32.90	35.79	32.66	35.89
Load out chute	29.10	30.28	28.49	35.37	32.90	33.41	35.75	34.00

a NA, Not available. Animals had been euthanized or had died.

b ND, Not detected following 40 cycles.
